# Effects of arousal on cognitive control: empirical tests of the conflict-modulated Hebbian-learning hypothesis

**DOI:** 10.3389/fnhum.2014.00023

**Published:** 2014-01-30

**Authors:** Stephen B. R. E. Brown, Henk van Steenbergen, Tomer Kedar, Sander Nieuwenhuis

**Affiliations:** ^1^Cognitive Psychology Unit, Institute of Psychology, Leiden UniversityLeiden, Netherlands; ^2^Leiden Institute for Brain and CognitionLeiden, Netherlands

**Keywords:** conflict, Stroop effect, interference, arousal, pupillometry, accessory stimulus

## Abstract

An increasing number of empirical phenomena that were previously interpreted as a result of cognitive control, turn out to reflect (in part) simple associative-learning effects. A prime example is the proportion congruency effect, the finding that interference effects (such as the Stroop effect) decrease as the proportion of incongruent stimuli increases. While this was previously regarded as strong evidence for a global conflict monitoring-cognitive control loop, recent evidence has shown that the proportion congruency effect is largely item-specific and hence must be due to associative learning. The goal of our research was to test a recent hypothesis about the mechanism underlying such associative-learning effects, the conflict-modulated Hebbian-learning hypothesis, which proposes that the effect of conflict on associative learning is mediated by phasic arousal responses. In Experiment 1, we examined in detail the relationship between the item-specific proportion congruency effect and an autonomic measure of phasic arousal: task-evoked pupillary responses. In Experiment 2, we used a task-irrelevant phasic arousal manipulation and examined the effect on item-specific learning of incongruent stimulus–response associations. The results provide little evidence for the conflict-modulated Hebbian-learning hypothesis, which requires additional empirical support to remain tenable.

## INTRODUCTION

Cognitive control is required to flexibly adapt our behavior to situational demands. It refers to the human capability to obtain a desired outcome given conflicting options. Stopping for a red traffic light, choosing an apple over chocolate, or finishing a paper rather than sitting in the sun, are all examples of cognitive control. In laboratory settings cognitive control is often measured using congruency tasks such as the Stroop task (MacLeod, 1992). Participants in the Stroop task are required to name the printed color of a color word (e.g., the word *blue* written in black ink). To do so they need to suppress their habitual tendency to respond to the color word (blue) and instead respond to the demanded ink color (black). Participants lacking cognitive control would respond habitually to the stimulus, which is demonstrated by many patients with damage to their prefrontal cortex (Vendrell et al., 1995).

Botvinick and colleagues proposed the conflict-monitoring hypothesis to explain how our cognitive system detects situations in which cognitive control is required (Botvinick et al., 2001). They suggested that the anterior cingulate cortex (ACC) monitors the occurrence of conflict in information processing. When conflict is detected, compensatory adjustments in control are made by passing on information to brain systems responsible for the exertion of cognitive control. Numerous neuroimaging studies have provided support for the idea that the ACC responds to the occurrence of conflict and then recruits areas responsible for cognitive control, such as the prefrontal cortex (Botvinick et al., 2004).

Botvinick et al. (2001) suggested that the conflict-monitoring hypothesis can also explain a number of important behavioral phenomena, including the conflict-adaptation effect and the proportion congruency effect. The conflict-adaptation effect refers to the finding that the magnitude of behavioral interference effects in congruency tasks is influenced by the congruency of the previous trial (Gratton et al., 1992). When two consecutive incongruent trials are presented, the degree of interference is smaller for the second trial. For example, in a Stroop task, responses to incongruent stimuli are faster and more accurate when those stimuli are preceded by another incongruent stimulus rather than a congruent stimulus. According to the conflict-monitoring hypothesis, this conflict-adaptation effect reflects an adjustment of cognitive control, signaled on a trial-by-trial basis by the ACC. Conflict on the preceding trial thus leads to higher levels of control on the subsequent trial.

The proportion congruency effect refers to the finding that the proportion of incongruent stimuli influences the magnitude of the interference effect that is measured in congruency tasks. For example, in a Stroop task, blocks of trials with a high proportion of incongruent stimuli will be associated with a smaller Stroop effect than blocks of trials with a small proportion of incongruent stimuli (Logan and Zbrodoff, 1979; Jacoby et al., 2003). The conflict-monitoring hypothesis explains this reduced interference effect as the result of a general increase in cognitive control, brought about by the frequent occurrence of conflict-inducing incongruent trials. A comparable hypothesis has been developed by Jacoby and colleagues (Jacoby et al., 2003; Blais et al., 2007; Bugg et al., 2008; Bugg et al., 2011a), who suggest that control is exerted at the item-level by attenuating word reading for items that are presented mostly incongruently, while boosting word reading for items that are presented mostly congruently.

However, recent evidence appears to contradict the notion that the behavioral phenomena discussed above can be fully explained by a global, pro-active control mechanism. Increasing evidence suggests that both the conflict-adaptation effect (e.g., Mayr et al., 2003; Nieuwenhuis et al., 2006) and the proportion congruency effect (Jacoby et al., 2003; Notebaert and Verguts, 2007; Bugg et al., 2008; Schmidt and Besner, 2008; Blais and Bunge, 2010) can be explained, at least in part, as a result of simple associative learning. For example, Blais and Bunge (2010) used a modified Stroop task, in which the global (or *list-level*) proportion congruency in a block of trials was either 30, 50, or 70%. Importantly, embedded within each block was an item-level proportion-congruent manipulation. The 30% block contained two items (i.e., color names) that were congruent on 10% of the trials and two items that were congruent on 50% of the trials. In the 50% block, all items were congruent on 50% of the trials. And in the 70% block, two items were congruent on 50% of the trials and two items were congruent on 90% of the trials. When comparing the 50% conditions from each block, Blais and Bunge found no proportion congruency effect. That is, when item-specific proportion congruency (ISPC) was held constant, list-level proportion congruency did not modulate the Stroop effect. In contrast, there were clear ISPC effects within the 30 and 70% blocks: items that occurred in incongruent form more often were associated with a smaller Stroop effect. Thus, in Blais and Bunge’s study, the proportion congruency effect seemed to be driven entirely by ISPC effects, thus undermining explanations in terms of global changes in control (but see Bugg et al., 2011b, for a demonstration of list-level control effects). A straightforward explanation of the ISPC effect is that it reflects the strengthening of incongruent stimulus–response associations as a function of the number of encounters with a particular item: the stronger the learned association between stimulus and response, the faster the reaction times (RTs; Schmidt and Besner, 2008).

**FIGURE 1:**
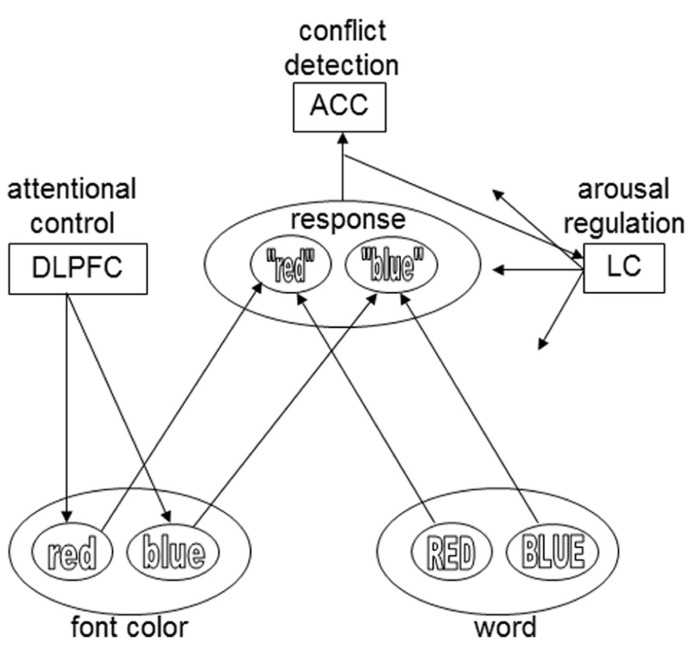
**The presentation of an incongruent Stroop stimulus (e.g., the word BLUE printed in red) causes conflict, which is detected by a conflict-monitoring system (ACC).**The ACC activates the LC, which releases norepinephrine throughout the cortex. Norepinephrine strengthens Hebbian learning for active representations. During subsequent presentations of the specific stimulus type, conflict is reduced and reaction time is shorter. The DLPFC influences the input layers, based on task demands: in this example, font color is the relevant stimulus dimension, and the task demand unit therefore biases that input layer. For the sake of simplicity, only two colors and response options are plotted here. The arrows from the LC module in this model exemplify the widespread projections of the LC. However, in the model of Verguts and Notebaert (2008, 2009) conflict-induced release of norepinephrine only modulates the connections between input and task demand units – an assumption that was modified in later work by these authors (e.g., Braem et al., 2011).

The goal of the current research was to test a recent hypothesis about the mechanism underlying associative-learning effects in conflict paradigms like the Stroop task: the conflict-modulated Hebbian-learning hypothesis, proposed by Verguts and Notebaert (2008, 2009). They proposed that conflict, such as experienced on an incongruent Stroop trial, triggers a phasic arousal response. This increase in arousal increases the rate of Hebbian learning of all representations that are active at the same time, thus enhancing learning of the association between the stimulus and relevant task (i.e., attentional control) representation. The strengthening of these associations then allows faster responses the next time they are activated. More formally, Verguts and Notebaert (2008, 2009) propose that the ISPC effect follows from a Hebbian-learning rule with a variable learning-rate parameter that is proportional to the degree of conflict (and consequent arousal) experienced on each trial. Thus, in a Stroop task a color word, say “red” printed in blue ink, may be presented (**Figure 1**). In accordance with the conflict-monitoring hypothesis (Botvinick et al., 2001), a conflict-monitoring system detects the conflict evoked by this incongruent stimulus. Contrary to the conflict-monitoring theory, however, conflict-mediated arousal then increases Hebbian learning, updating the weights of the connections between stimulus and task-demand representations. The next time the word red is presented in blue, the corresponding connections are strengthened and Stroop interference decreases. The more frequent a particular item, the more pronounced the improvement in performance associated with that item.

Verguts and Notebaert (2009) have also proposed a neural mechanism for conflict modulated Hebbian-learning (**Figure 1**). Similar to the conflict-monitoring hypothesis, they suggest that conflict is detected by the ACC. The ACC then triggers a phasic response of the locus coeruleus (LC), a small noradrenergic brainstem nucleus with a major role in regulating arousal, through its widespread ascending projections throughout the brain (Sara, 2009). LC activation results in the global release of the neuromodulator norepinephrine (NE), which is known to strengthen Hebbian learning (reviewed in Berridge and Waterhouse, 2003; Bouret and Sara, 2005; Nieuwenhuis, 2011).

Although the conflict-modulated Hebbian-learning hypothesis is in line with neurophysiological and anatomical findings (Verguts and Notebaert, 2009), there is very little empirical evidence that item-specific associative learning in cognitive control tasks is indeed mediated by phasic arousal (van Bochove et al., 2013). In the current study, we investigated this hypothesis in two experiments. In Experiment 1, we took a correlational approach and examined in detail the relationship between the ISPC effect and an autonomic measure of phasic arousal: task-evoked pupillary responses. In Experiment 2, we used a phasic arousal manipulation and examined the effect of arousal on item-specific learning of stimulus-response associations in a cognitive control task.

## EXPERIMENT 1

We adapted the Stroop task experiment conducted by Blais and Bunge (2010), **Figure 1**). Participants performed two blocks of Stroop trials with 240 trials each. List-level and item-level proportion congruency were manipulated: one block consisted of item types that were congruent on 10 or 50% of the trials (list-level congruency = 30%) and the other block consisted of item types that were congruent on 50 or 90% of the trials (list-level congruency = 70%). Throughout the experiment we measured task-evoked pupil dilation, a broadly accepted measure of phasic autonomic arousal (Bradshaw, 1967; Kahneman, 1973; Bradley et al., 2008; Nieuwenhuis et al., 2011). Previous research has found increased pupil dilations on incongruent trials compared to congruent trials in the Stroop task (Siegle et al., 2004; Laeng et al., 2011) and similar paradigms (van Steenbergen and Band, 2013), suggesting that pupil diameter is sensitive to conflict. The conflict-modulated Hebbian learning hypothesis suggests that participants with a larger pupillary arousal response to conflict, as indexed by the modulation of pupil dilation by congruency, should show a larger ISPC effect.

### METHODS

#### Participants

Twenty-four non-color blind participants (2 males), aged 18–27 took part in a single 1.5-h experimental session in return for course credit or €10. Participants signed informed consent prior to their inclusion in the study.

#### Stimuli and task

Participants performed a version of the Stroop task adapted from Blais and Bunge (2010), implemented in E-Prime (Psychology Software Tools, Sharpsburg, PA, USA). Each trial started with a fixation stimulus that was presented for 2.5, 3.0, or 3.5 s. To preclude luminance differences between the fixation stimulus and the subsequent target stimulus, we created fixation stimuli by scrambling pixels of all four target colors used in a task block. A Stroop stimulus, presented for 1,500 ms, followed the fixation, after which the next trial started. Participants were instructed to respond to the color of this stimulus, not to the color word presented on the screen. To help participants maintain the stimulus–response mappings, throughout the experiment 4 color patches were located at the bottom of the screen. These color patches corresponded spatially with the d, f, j, and k keys on a standard QWERTY keyboard, and represented the stimulus colors presented in that block. We used a subset of 8 colors from the 12 used by Blais and Bunge (2010). In one block, a color set of pink (RGB values 255, 192, 203), green (000, 176, 080), brown (139, 069, 019), and yellow (255, 255, 000) was used; in the other block blue (000, 112, 192), red (255, 000, 000), white (250, 250, 250), and purple (112, 048, 160) were used. Within a block, two sets of stimuli were grouped together; for example, green was presented in either green or pink, but never in yellow or brown. Participants were instructed verbally to fixate the center of the screen throughout each trial.

#### Design and procedure

Following Blais and Bunge (2010), we manipulated the ISPC within task blocks and the list-level proportion congruency between task blocks. In one block, two color items were congruent in 50% of the trials, while the other items were congruent in 10% of the trials, resulting in a list-level proportion congruency of 30%. In the other block, two color items were congruent in 50% of the trials, while the other items were congruent in 90% of the trials, resulting in a list-level proportion congruency of 70%. For the sake of brevity, we will use codes like 30/10 in the description of the results to indicate list-level proportion congruency (30%) and then item-level proportion congruency (10%). Both color sets and proportion congruency order were counterbalanced across participants. Each block consisted of 240 trials, yielding a total of 480 trials; participants could take a short break halfway through a block.

Prior to each experimental block, participants received on-screen instructions and performed 24 practice trials to familiarize themselves with the stimulus-response mappings. Each color was presented six times in the form of a large rectangle in the middle of the screen. Participants had to respond to the color of the rectangle. If following the practice trials participants indicated that they had not correctly learned the stimulus–response mappings, they received another practice block of 24 trials. Participants then proceeded to the experimental condition.

During the experiment, pupil diameter was measured continuously. The experiment was conducted in a slightly dimmed room.

#### Pupil data acquisition and analysis

We recorded pupil diameter at 60 Hz using a Tobii T120 eye tracker monitor (Tobii Technology, Stockholm, Sweden), integrated into a 17″ TFT monitor. Participants were seated at about 60 cm from the screen. Pupil measurements were made without the use of a head rest, because the Tobii T120 eyetracker is not sensitive to head movements (user manual; Tobii, Danderyd, Sweden). We analyzed the pupil data in Brain Vision Analyzer with custom-made macros. Artifacts and blinks were adjusted by linear interpolation. Extremely unreliable interpolated data points (i.e., < 30% valid data points in the interval of interest) were excluded from analyses. Pupil dilation was defined in the averaged waveform as the peak pupil diameter during the period from 550 to 2500 ms following stimulus onset, relative to a 200-ms prestimulus baseline.

### RESULTS

#### Behavior

**FIGURE 2:**
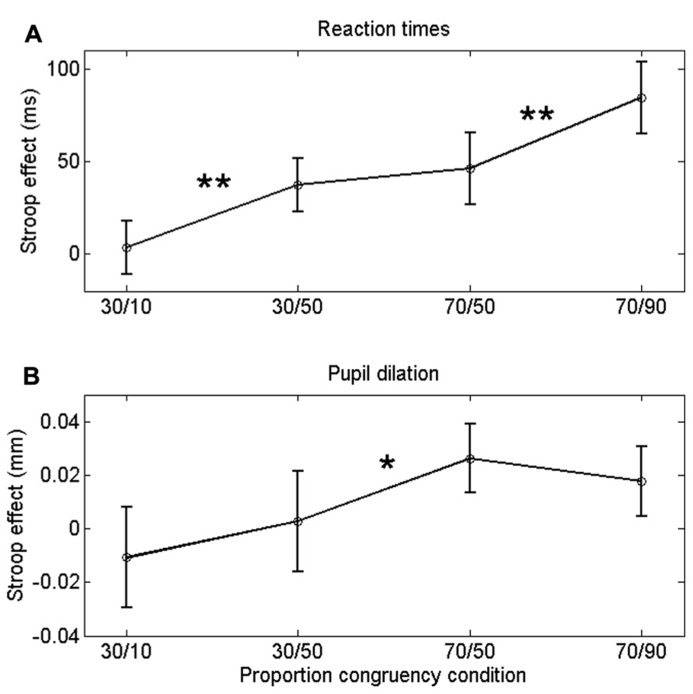
**Congruency (Stroop) effect on RT (A) a and pupil dilation **(B)** for each list-level/item-specific proportion congruency.** Asterisks indicate significant differences: **p < 0.01; *p < 0.05. Error bars are based on within-subjects error (Masson and Loftus, 2003).

**Table 1** displays the mean correct RTs for each task condition. Mean Stroop effects are plotted in **Figure 2A**. Because item and list proportion congruency were not varied in an orthogonal fashion, a traditional factorial analysis is difficult to interpret. Following the approach by Blais and Bunge (2010), we analyzed the ISPC effect independently of list-level proportion congruency by running two separate 2 (congruency) × 2 (item proportion congruency) analyses of variance (ANOVAs).

**Table 1:** Mean correct reaction times (standard deviation) for each task condition.

	30/10	30/50	70/50	70/90
Congruent	626 (56)	608 (60)	618 (59)	607 (51)
Incongruent	630 (63)	645 (71)	664 (77)	691 (109)

Incongruent trials were associated with longer RTs than congruent trials, both in the 30% block, *F*(1,23) = 11.8, *p *= 0.002, and in the 70% block, *F*(1,23) = 32.8, *p *< 0.0005. Importantly, the Stroop effect was larger in the 30/50 condition (37 ms) than in the 30/10 condition (4 ms), *F*(1,23) = 11.8, *p *= 0.002. Similarly, the Stroop effect was larger in the 70/90 condition (84 ms) than in the 70/50 condition (46 ms), *F*(1,23) = 8.3, *p *= 0.009. Thus, participants showed robust ISPC effects. In contrast, the difference in Stroop effects between the 30/50 (37 ms) and 70/50 (46 ms) conditions was not significant, *t*_23_ = 1.2, *p *= 0.25, indicating that RTs were not substantially influenced by list-level proportion congruency.

Because mean error rates were very low and did not differ much between congruent (2%) and incongruent trials (3%), we did not analyze them further.

#### Pupillometry

**FIGURE 3:**
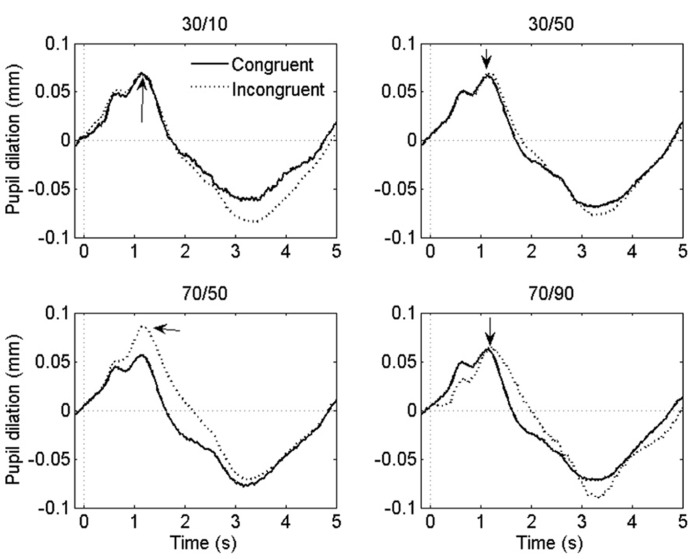
**Grand-average pupil waveforms in each task condition.** Time = 0 ms indicates the onset of the stimulus. The arrows indicate the dilatory peaks the analysis was based on.

Grand-average pupil waveforms are plotted in **Figure 3**.

If the ISPC effect described above is driven by conflict-induced arousal, then pupil dilations should demonstrate a similar sensitivity to task conditions as RT. Mean pupil Stroop effects are plotted in **Figure 2B**; as is clear from that graph, the pupil data do not match the RT data in **Figure 2A**. We analyzed the pupil data in the same manner as the behavioral data, by running two separate 2 (congruency) × 2 (item proportion congruency) ANOVAs. In the 30% block, there was no reliable difference in pupil dilation between congruent (0.094 mm) and incongruent trials (0.090 mm), *F*(1,23) = 0.43,* p *= 0.52. In the 70% block, we found a trend in the expected direction: incongruent stimuli elicited larger dilations (0.106 mm) than congruent stimuli (0.084 mm), *F*(1,23) = 3.69,* p = *0.07. Importantly, there was no reliable difference in pupil Stroop effects between the 30/50 (-0.011 mm) and the 30/10 (0.002 mm) conditions, *F*(1,23) = 1.1, *p *= 0.52; and also no reliable difference between the 70/50 (0.026 mm) and the 70/90 (0.018 mm) conditions, *F*(1,23) = 0.7, *p *= 0.69. So, unlike the behavioral data, the pupil-dilation data did not show evidence of a robust ISPC effect. Furthermore, the difference in pupil Stroop effect between the 30/50 (0.003 mm) and 70/50 (0.026 mm) conditions was significant, *t*_23_ = 2.3, *p *= 0.03, indicating a list-level proportion congruency effect, again unlike in the behavioral data.

To examine the robustness of these results, we carried out some additional analyses, in which we focused on the frequently observed coupling between baseline pupil diameter and pupil dilations. First, because pupil dilations (averaged across all conditions) were modulated by individual differences in baseline pupil diameter (dilations on incongruent trials: *r *= 0.42, *p *= 0.04; on congruent trials *r *= 0.25, *p *= 0.23), we analyzed the data separately for the 12 participants with the smallest and the 12 with the largest baseline pupil: in both groups, we found a pattern similar to the grand average in **Figure 2B**. Indeed, only 3 out of 24 participants showed a pattern of pupil Stroop effects that was monotonically increasing like the RT Stroop effects.

Second, we checked if the list-level effect in the pupil-dilation data was accompanied by and perhaps caused by a difference between blocks in baseline pupil diameter. Such a difference in baseline pupil might reflect the difference in task difficulty associated with different proportions of incongruent trials. However, a *t*-test showed no reliable difference in baseline diameters between the 30% (3.21 mm) and the 70% (3.22 mm) blocks, *t*_23_ = 0.39, *p *= 0.70. Together, these control analyses suggest that the pattern of pupil-dilation Stroop effects cannot be explained by differences in baseline pupil.

#### Behavior-pupil correlations

**FIGURE 4:**
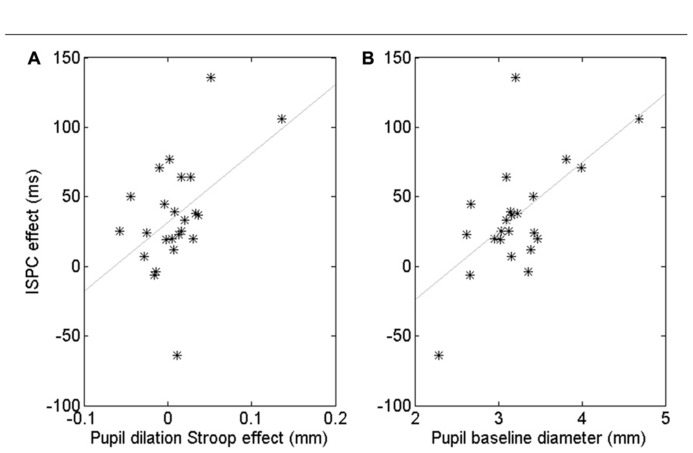
**Correlation between the pupil-dilation Stroop effect and the behavioral ISPC effect (A).** Correlation between pupil baseline diameter and the ISPC effect **(B)**. Each star represents one subject.

Although the pupil data showed no robust ISPC effect, there were substantial individual differences. To gain insight in these individual differences, we computed a number of correlations. First, we quantified the behavioral ISPC effect for every participant by averaging the item-related difference in Stroop effects in each block, that is [(Stroop3050 - Stroop3010) + (Stroop7090 - Stroop7050)]/2. This ISPC effect reflects the sensitivity of each participant to the item-level proportion congruency, with large ISPC scores indicating high sensitivity. We also calculated for each participant the average pupil Stroop effect to index the effect of conflict on the arousal system, the process hypothesized by Verguts and Notebaert (2008) to underlie the ISPC effect. We then investigated whether participants with a larger pupil Stroop effect also showed a larger ISPC effect. The Pearson correlation was *r* = 0.47, *p* = 0.020. However, **Figure 4A** suggests that this significant correlation may have been driven by a few outliers. Spearman’s rank correlation, which is less sensitive to (univariate) outliers than Pearson’s coefficient, was marginally significant, **ρ = 0.38, *p *= 0.07, suggesting some evidence that people whose pupil diameter is more sensitive to Stroop conflict tend to have a larger ISPC effect.

Next, we examined if differences in overall baseline pupil diameter were also predictive of a participant’s behavioral ISPC effect. Indeed, we found a significant Pearson correlation, *r* = 0.60, *p* = 0.002, and Spearman rank correlation,**ρ**= 0.41, *p *< 0.05 (**Figure 4B**). The Pearson correlation remained significant after partialling out the contribution of the pupil-dilation Stroop effect: *r* = 0.51, *p *= 0.01, suggesting that baseline pupil diameter explains unique variance in the ISPC effect. Indeed, step-wise regression analysis indicated that a model with both pupil-dilation Stroop effect and baseline pupil diameter as predictors explained the individual differences in the size of the ISPC effect better than a model with only the pupil-dilation Stroop effect as predictor, *F*_change_ = 0.013.

Finally, because pupil dilation showed only a modest Stroop effect (in the 70% block), we further investigated the sensitivity of pupil diameter to response conflict by correlating the Stroop effects in the pupil-dilation and behavioral data. Pooled across conditions these measures showed a large positive correlation (*r* = 0.62, *p* = 0.001). Significant positive correlations (*p*s < 0.05) were also found within the 30/50, 70/50, and 70/90 conditions, but not in the 30/10 condition, presumably because the Stroop effects in that condition were virtually absent (**Figure 2**). Thus, altogether the data indicate that pupil dilation reliably scaled with response conflict.

### DISCUSSION

The results of Experiment 1 provide mixed evidence for the conflict-modulated Hebbian learning hypothesis. Participants showed a strong ISPC effect: the observed list-level proportion congruency effect on Stroop interference was almost entirely due to differences in proportion congruency at the item-level, suggesting an important role for associative learning. However, the differences in Stroop effect between items were not mirrored by corresponding changes in pupil dilation; in contrast, pupil dilation showed a list-level effect and no ISPC effect. This is inconsistent with the hypothesis that associative learning effects in the Stroop task are modulated by conflict-induced arousal. Conversely, the behavior-pupil correlations did show some evidence for another key prediction of the conflict-modulated Hebbian learning hypothesis, namely that people whose pupil diameter is more sensitive to Stroop conflict (i.e., who exhibit more conflict-induced arousal) should have a larger ISPC effect. Finally, baseline pupil diameter, measured across the whole experiment, also predicted the behavioral ISPC effect, accounting for significant variance over and above that explained by conflict-induced pupil dilation.

These results are broadly consistent with a recent study in which pupil dilation and baseline pupil together predicted learning rate in a predictive-inference task (Nassar et al., 2012). In that study, the amplitudes of baseline pupil and pupil dilation correlated with distinct measures of uncertainty (as defined by a normative model) that together indicated the influence that new data should have on existing beliefs. Although most of the results concerned relationships across single trials within participants, Nassar and colleagues also found evidence that participants with a larger average pupil size attributed more weight to incoming data, i.e., exhibited larger learning rates. That result, albeit in a different context, mirrors our finding that participants with a larger baseline pupil showed enhanced learning of stimulus–response associations. Furthermore, Silvetti et al. (2013) reported a correlation between pupil diameter and learning rate in a probabilistic learning task. The observed relationship between individual differences in pupil metrics and the ISPC effect also seems broadly consistent with the hypothesized role of the LC-NE system in associative learning in cognitive control contexts (Verguts and Notebaert, 2009). Although the evidence is still preliminary, neurophysiological (Aston-Jones and Cohen, 2005), neuroimaging (Murphy et al., 2011; Murphy et al., in press), anatomical (Nieuwenhuis et al., 2011), pharmacological (Phillips et al., 2000), and behavioral evidence (Gilzenrat et al., 2010; Jepma and Nieuwenhuis, 2011) suggests that pupil diameter is a correlate of LC-NE activity: baseline pupil diameter of tonic LC activity and task-evoked pupil dilations of phasic LC activity. On this assumption our results are consistent with empirical evidence and models that posit an important role for both tonic and phasic LC activity in learning (Bouret and Sara, 2005; Yu and Dayan, 2005; Nieuwenhuis, 2011).

## EXPERIMENT 2

According to Verguts and Notebaert (2009), the conflict-modulated Hebbian-learning hypothesis predicts that arousal-inducing but task-irrelevant stimuli should lead to enhanced learning of the association between accompanying task-relevant stimuli and responses. Experiment 2 was designed as a first test of this important prediction, using a task-independent manipulation of phasic arousal. We used a conflict task in which specific incongruent stimuli were frequently accompanied by a task-irrelevant loud auditory tone. Such an accessory stimulus (AS) is known to decrease RTs to the task-relevant stimulus (e.g., Bernstein, 1970), and increase the weight of new data (Nassar et al., 2012), presumably through a phasic burst of arousal (Sanders, 1983; Hackley and Valle-Inclán, 2003; Jepma et al., 2009). In Experiment 2 we were primarily interested in the progression of RTs on incongruent trials without an AS: the conflict-modulated Hebbian-learning hypothesis predicts a steeper learning rate (i.e., a faster decrease in RT) for stimulus-response associations that were frequently paired with an AS, compared to associations that were never paired with an AS.

We expected any existing arousal effect on learning to be small in size. To be able to detect such a small effect we designed a task in which RT differences between incongruent stimulus–response associations were minimal. The task was a four-choice Simon task, in which participants were required to classify stimulus identity by pressing 1 of 4 spatially arranged buttons, while trying to suppress the urge to respond according to the task-irrelevant stimulus location. Previous research has reported a conflict-based arousal effect (van Steenbergen and Band, 2013) and a typical proportion congruency effect (Borgmann et al., 2007) in this type of task, suggesting that performance in this task is sensitive to the same type of learning as performance in the Stroop task.

### METHODS

#### Participants

Twenty participants (five males), aged 19–28, took part in a single 30-min experimental session in return for course credit or €3.50. Participants signed informed consent prior to inclusion in the study.

#### Stimuli and task

**FIGURE 5:**
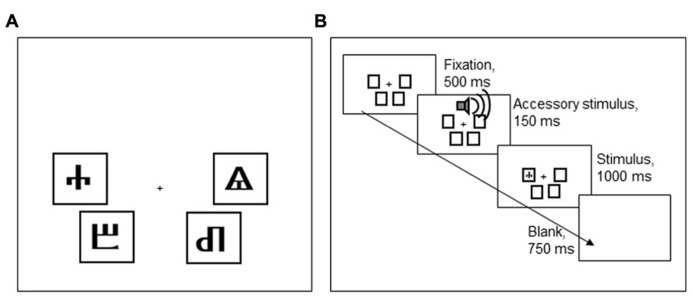
**(A)** An overview of the four Glagolitic stimuli and their congruent locations on the screen. **(B)** Order of task events in Experiment 2.

Participants performed a Simon task, implemented in E-Prime (Psychology Software Tools, Sharpsburg, PA, USA). Each trial started with a fixation stimulus that was presented for 500 ms. The fixation stimulus was followed by an imperative stimulus, presented for 1,000 ms, and a blank screen, presented for 750 ms. The imperative stimulus, one of four Glagolitic characters, could appear in four positions on the screen, indicated by black frames (see **Figure 5**). The participant’s task was to classify the stimulus identity by pressing one of four keys on a QWERTY keyboard (a, z, k, m). To make stimulus location, the task-irrelevant stimulus dimension, more salient, the four keys had a similar spatial configuration as the four screen locations where stimuli could appear.

To learn the stimulus-response mappings, participants first performed 80 practice trials in which stimuli were presented in the center of the screen, and feedback on response accuracy was presented after every trial. Following this block, participants performed 16 additional practice trials in which stimuli appeared in one of the four positions on the screen, as in the experimental block. Participants’ accuracy was presented at the end of this block. After ensuring the participant fully comprehended the task, the experimental block was commenced. In total, 720 experimental trials were presented in four blocks of 180 trials: 360 trials were congruent (i.e., stimulus and response locations matched) and 360 trials were incongruent (non-matching stimulus and response locations). Every stimulus was presented 180 times: in incongruent trials, it appeared with equiprobability in one of the three incongruent locations.

Two of the four stimuli were frequently accompanied by a loud AS tone [800 Hz, 77 dB(A), 150 ms] that started 30 ms prior to the onset of the imperative stimulus. The AS accompanied these stimuli on 50% of the incongruent trials, never on congruent trials. The other two stimuli were never accompanied by an AS. The stimuli that could be accompanied by an AS were counterbalanced across participants, so that either the stimuli that were congruent in the lower left and upper right, or those that were congruent in the lower right and upper left locations could be associated with an AS (cf. **Figure 5A**). Participants were told that the tones were unrelated to the task and that they should try to ignore the sounds.

### RESULTS

As expected, congruent trials were associated with shorter RTs (562 ms) than incongruent no-AS trials (588 ms), *F*(1,19) = 441, *p *< 0.0005, as well as lower error rates (3.9%) than incongruent no-AS trials (11.2%), *F*(1,19) = 81, *p* < 0.0005.

#### Manipulation check

To test whether our manipulation of arousal was successful, we computed the AS effect: the difference in correct RT between AS trials and no-AS trials. RTs on incongruent AS trials (620 ms) were significantly shorter than RTs on incongruent no-AS trials (635 ms), *F*(1,19) = 7.43, *p* = 0.01, yielding a typical AS effect of 15 ms. Incongruent AS trials were associated with a marginally higher error rate (13.8%) than incongruent no-AS trials (12.2%), but this difference was not reliable *F*(1,19)**= 1.20, *p *= 0.29.

#### AS effect on learning rate

To examine the effect of arousal on learning rate, we compared the progression of RTs on incongruent no-AS trials that were frequently paired with an AS with the progression of RTs on incongruent no-AS trials that were never paired with an AS. We refer to these categories of trials as AS^+^ and AS^-^. For each participant, the 90 AS^+^ trials and 180 AS^-^ trials were equally divided in five chronological bins. Before averaging the RTs in each bin, RT outliers and incorrect trials were replaced by an RT that was interpolated by computing the average RT of trials *n *- 1 and *n *+ 1 of the corresponding trial type (AS^+^ or AS^-^). The resulting time series were averaged across participants

**FIGURE 6:**
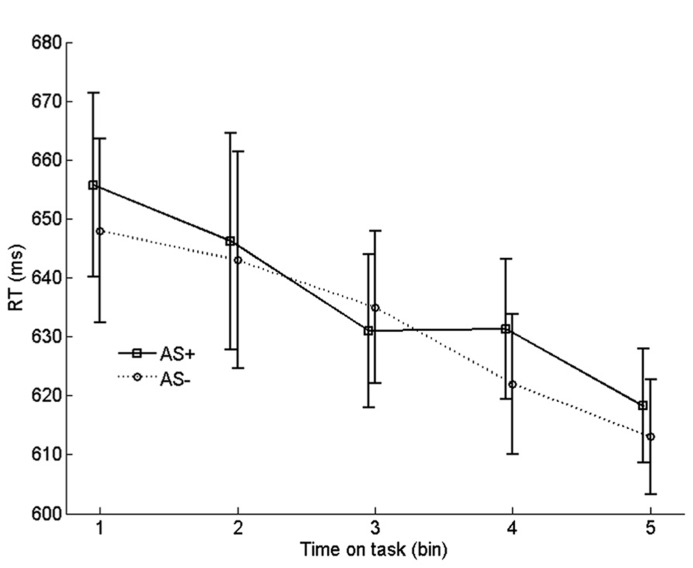
**Binned RTs on no-AS trials for stimuli that were frequently accompanied by an accessory stimulus (AS^+^) vs. stimuli that were never accompanied by an AS (AS^-^).** Error bars are based on within-subjects error terms associated with each of the five pairwise comparisons (Masson and Loftus, 2003).

As shown in **Figure 6**, incongruent RTs monotonically decreased with time on task (bin), reflecting (at least in part) the gradual strengthening of learned stimulus–response associations in the face of conflict. Importantly, there was no systematic difference in learning rate between AS^+^ and AS^-^ trials. To test this, we quantified the learning rate for each trial type as the average RT of bin 1 minus the average RT of bin 5. Indeed, learning rate on AS^+^ trials (37 ms) did not differ significantly from the learning rate on AS^-^ trials (35 ms), *t*_19_ = 0.19, *p *= 0.86^1^. A similar analysis with learning rate quantified as the difference between bin 1 and bin 3 also yielded a non-significant difference between AS^+^ and AS^-^ trials, *p* = 0.32. Furthermore, comparisons, for each bin, between AS^+^ and AS^-^ trials yielded no significant differences in RT, all *p*s > 0.26. These findings suggest that there was no robust effect of arousal on learning of task-relevant stimulus-response associations.

### DISCUSSION

In Experiment 2 we found no evidence that phasic arousal enhances learning of incongruent stimulus–response associations. Although the manipulation of phasic arousal was successful, as indicated by a robust AS effect on RT, the monotonic decrease in RTs on no-AS trials across the experiment was virtually identical for AS^+^ and AS^-^ trials. This is inconsistent with the conflict-modulated Hebbian-learning hypothesis (Verguts and Notebaert, 2008, 2009), which suggests that arousal should precipitate the gradual strengthening of stimulus-response associations. Jacoby et al. (2003) reported evidence that the ISPC effect can emerge rapidly, suggesting that in our experiment associative learning might already have occurred within the course of our first bin. However, we also found no evidence for a learning effect in the first couple of bins: if anything, RTs on AS^+^ trials were slower than RTs on AS^-^ trials.

A limitation of Experiment 2 is that we did not collect a physiological measure of tonic arousal level, such as baseline pupil size. An interesting question for future research is whether the predicted learning effect might be present for a subgroup of participants with lower baseline arousal. This question is inspired by the study of Nassar et al. (2012), in which the direction of AS-induced learning effects was dependent on *trial-specific* changes in baseline pupil size.

## GENERAL DISCUSSION

An increasing number of empirical phenomena that were previously interpreted as a result of cognitive control, turn out to reflect (in part) simple memory and learning mechanisms (Jacoby et al., 2003; Mayr et al., 2003; Schneider and Logan, 2005). A prime example is the proportion congruency effect, the finding that interference effects, such as the Stroop effect, decrease as the proportion of incongruent stimuli increases. While this was previously regarded as strong evidence for a global conflict monitoring-cognitive control loop (Botvinick et al., 2001), recent evidence has shown that the proportion congruency effect is largely item-specific and must be due to cumulative associative learning. The goal of our research was to test a recent hypothesis about the mechanism underlying such associative-learning effects: the conflict-modulated Hebbian-learning hypothesis (Verguts and Notebaert, 2008, 2009), a computationally and neurobiologically grounded account which proposes that the effect of proportion congruency on associative learning is mediated by conflict-triggered phasic arousal responses. Our study provided the first direct empirical tests of the conflict-modulated Hebbian-learning hypothesis. In general, the results present some positive but mainly negative evidence for this account. We conclude that although Verguts and Notebaert’s hypothesis presents an elegant integrative account of conflict-related associative learning effects, it requires additional empirical support to remain tenable.

In Experiment 1, we found that participants who exhibited more conflict-induced arousal, as indexed by task-evoked pupillary responses, had a larger ISPC effect. This finding provides compelling support for the conflict-modulated Hebbian-learning hypothesis. However, a different analysis showed that the behavioral Stroop effect is sensitive to item-level proportion congruency, while pupil dilation showed a list-level effect and no ISPC effect. This is hard to reconcile with the hypothesis that associative learning effects reflected in item-specific Stroop effects were driven by conflict-induced arousal. Furthermore, baseline pupil diameter was an even stronger predictor of the behavioral ISPC effect than conflict-induced pupil dilation, suggesting that the ISPC effect reflects tonic more than phasic arousal. In Experiment 2, we found that a task-irrelevant phasic arousal manipulation did not affect item-specific learning of stimulus-response associations, even though the manipulation was clearly successful in modulating response speed. This finding refutes an important prediction of the conflict-modulated Hebbian-learning hypothesis.

The fact that we do not find unequivocal evidence for the conflict-modulated Hebbian learning hypothesis in two experiments suggests that certain assumptions of the model may have to be revised. One possibility implied by our findings is that the relationship between conflict and associative learning is not mediated by LC-induced arousal. However, this assumption (Verguts and Notebaert, 2009) is supported by various lines of evidence: the anatomical connection between the ACC and the LC is well-established (Aston-Jones and Cohen, 2005), and the noradrenergic system is known to be important for learning (Yu and Dayan, 2005; Nieuwenhuis, 2011; Eldar et al., 2013). Furthermore, it is known that conflict leads to arousal (Berlyne et al., 1966; Laeng et al., 2011; van Steenbergen and Band, 2013) and that arousal is important for learning (Berlyne, 1957; Nassar et al., 2012). Nonetheless, there are indications that the relationship between conflict and learning rate may be (in part) mediated by other neuromodulator systems, such as the cholinergic system (Doya, 2002) and the dopaminergic system (van Bochove et al., 2013) suggest that dopamine may be involved in conflict tasks.

Alternatively, the model of Verguts and Notebaert (2008) could be misspecified at a more fundamental level. For example, the ISPC effect may not be related to conflict. However, that seems unlikely in the face of data (e.g., Crump et al., 2008; for reviews, see Bugg and Crump, 2012; Schmidt, 2013) that conflict does seem to be crucial for the ISPC effect. Furthermore, if conflict would not be relevant, then arguably the effect of item frequency on RT should be similar on congruent and incongruent trials, which is often not the case (see, e.g., **Table 1**; Crump et al., 2008; Blais and Bunge, 2010). Accordingly, if conflict is kept constant in the Hebbian-learning model of Verguts and Notebaert (2008), the model does not show an ISPC effect. These arguments suggest that conflict detection is essential for the ISPC effect.

It is also possible that conflict-modulated associative learning occurs not just between stimulus and task-demand representations, as in the model of Verguts and Notebaert (2008), but also between stimulus and response representations. Incorporating that assumption in the conflict-modulated Hebbian-learning account would unify the contingency account of the ISPC effect (Schmidt and Besner, 2008), which emphasizes learning of stimulus-response associations, and the item-specific control account (Bugg et al., 2008; Blais and Bunge, 2010), which assumes a major role for learning of stimulus-attention associations in causing the ISPC effect. A recent review reports evidence supporting both of these types of learning (Bugg and Crump, 2012). Indeed, in more recent work Verguts and Notebaert have proposed that conflicts also modulates stimulus-response associations (Braem et al., 2011; cf. Hommel et al., 2004). However, it is unlikely that this additional assumption can account for the current results.

To conclude, although important progress has been made in understanding the constituent components of proportion congruency effects (Bugg and Crump, 2012), much work remains to be done to elucidate the neurocognitive mechanisms underlying these effects. An important advantage of the model by Verguts and Notebaert (2008) is that it is computationally explicit, unlike some other models of the ISPC effect (but see Blais et al., 2007). This should allow validation of the current predictions, as well as facilitate the generation of new predictions, to be tested in future empirical research.

## Conflict of Interest Statement

The authors declare that the research was conducted in the absence of any commercial or financial relationships that could be construed as a potential conflict of interest.

## Footnotes

1 A Bayesian *t*-test comparing these learning rates yielded a JZS Bayes factor (Rouder etal., 2009) of 5.76, indicating that the data are 5.76 times more likely under the null hypothesis (i.e., no difference) than under the hypothesis suggested by the model of Verguts and Notebaert (2008, 2009).

## References

[B1] Aston-Jones G. Cohen J. D. (2005). An integrative theory of locus coeruleus-norepinephrine function: adaptive gain and optimal performance. *Annu. Rev. Neurosci.* 24 167–202 10.1037/h0041135 16022602

[B2] Berlyne D. E. (1957). Uncertainty and conflict: a point of contact between information-theory and behavior-theory concepts. *Psychol. Rev.* 64 329–339 10.1037/h0041135 13505970

[B3] Berlyne D. E. Borsa D. M. Hamacher J. H Koenig I. D. V. (1966). Paired-associate learning and the timing of arousal. *J. Exp. Psychol.* 72 1–6 10.1037/h0023325 5967727

[B4] Bernstein I. H. (1970). Can we see and hear at the same time? Some recent studies of intersensory facilitation of reaction time. *Acta Psychol.* 33 21–35 10.1016/0001-6918(70)90119-8 5445965

[B5] Berridge C. W. Waterhouse B. D. (2003). The locus coeruleus-noradrenergic system: modulation of behavioral state and state-dependent cognitive processes. *Brain Res. Rev.* 42 33–84 10.1016/S0165-0173(03)00143-7 12668290

[B6] Blais C. Bunge S. (2010). Behavioral and neural evidence for item-specific performance monitoring. *J. Cogn. Neurosci.* 22 2758–2767 10.1162/jocn.2009.21365 19925177

[B7] Blais C. Robidoux S. Risko E. F. Besner D. (2007). Item-specific adaptation and the conflict-monitoring hypothesis: a computational model. *Psychol. Rev.* 114 1076–1086 10.1037/0033-295X.114.4.1076 17907873

[B8] Botvinick M. Braver T. S. Barch D. M. Carter C. S. Cohen J. D. (2001). Conflict monitoring and cognitive control. *Psychol. Rev.* 108 624–652 10.1037/0033-295X.108.3.624 11488380

[B9] Botvinick M. Cohen J. D. Carter C. S. (2004). Conflict monitoring and anterior cingulated cortex: an update. *Trends Cogn. Sci.* 8 539–546 10.1016/j.tics.2004.10.003 15556023

[B10] Borgmann K. W. Risko E. E. Stolz J. A. Besner D. (2007). Simon says: reliability and the role of working memory and attentional control in the simon task. *Psychon. Bull. Rev.* 14 313–319 10.3758/BF03194070 17694919

[B11] Bouret S. Sara S. J. (2005). Network reset: a simplified overarching theory of locus coeruleus noradrenaline function. *Trends Neurosci.* 28 574–582 10.1016/j.tins.2005.09.002 16165227

[B12] Bradley M. M. Miccoli L. Escrig M. A. Lang P. J. (2008). The pupil as a measure of emotional arousal and autonomic activation. *Psychophysiology* 45 602–607 10.1111/j.1469-8986.2008.00654.x 18282202 PMC3612940

[B13] Bradshaw J. (1967). Pupil size as a measure of arousal during information processing. *Nature* 216 515–516 10.1038/216515a0 6057275

[B14] Braem S. Verguts T. Notebaert W. (2011). Conflict adaptation by means of associative learning. *J. Exp. Psychol. Hum. Percept. Perform.* 37 1662–1666 10.1037/a0024385 21728466

[B15] Bugg J. M. Crump J. C. (2012). In support of a distinction between voluntary and stimulus-driven control: a review of the literature on proportion congruent effects. *Front. Psychol.* 3:367 10.3389/fpsyg.2012.00367 PMC3459019 23060836

[B16] Bugg J. M. Jacoby L. L. Chanani S. (2011a). Why it is too early to lose control in accounts of item-specific proportion congruency effects. *J. Exp. Psychol. Hum. Percept. Perform.* 37 844–859 10.1037/a0019957 20718569

[B17] Bugg J. M. McDaniel M. A. Scullin M. K. Braver T. S. (2011b). Revealing list-level control in the Stroop task by uncovering its benefits and a cost. *J. Exp. Psychol. Hum. Percept. Perform.* 37 1595–1606 10.1037/a0024670 21767049 PMC3609544

[B18] Bugg J. M. Jacoby L. L. Toth J. P. (2008). Multiple levels of control in the stroop task. *Mem. Cognit.* 36 1484–1494 10.3758/MC.36.8.1484 PMC2682765 19015507

[B19] Crump M. J. C. Vaquero J. M. M. Milliken B. (2008). Context-specific learning and control: the role of awareness, task-relevance, and relative salience. *Conscious. Cogn.* 17 22–36 10.1016/j.concog.2007.01.004 17349805

[B20] Doya K. (2002). Metalearning and neuromodulation. *Neural Netw.* 15 495–506 10.1016/S0893-6080(02)00044-8 12371507

[B21] Eldar E. Cohen J. D. Niv Y. (2013). The effects of neural gain on attention and learning. *Nat. Neurosci.* 16 1146–1156 10.1038/nn.3428 23770566 PMC3725201

[B22] Gilzenrat M. S. Nieuwenhuis S. Jepma M. Cohen J. D. (2010). Pupil diameter tracks changes in control state predicted by the adaptive gain theory of locus coeruleus. *Cogn. Affect. Behav. Neurosci.* 10 252–269 10.3758/CABN.10.2.252 20498349 PMC3403821

[B23] Gratton G. Coles M. G. H. Donchin E. (1992). Optimizing the use of information: strategic control of activation and responses. *J. Exp. Psychol. Gen.* 4 480–506 10.1037/0096-3445.121.4.480 1431740

[B24] Hackley S. A. Valle-Inclán F. (2003). Which stages of processing are speeded by a warning signal? *Biol. Psychol.* 64 27–45 10.1016/S0301-0511(03)00101-7 14602354

[B25] Hommel B. Proctor R. W Vu K. -P. L. (2004). A feature-integration account of sequential effects in the Simon task. *Psychol. Res.* 68 1–17 10.1007/s00426-003-0132-y 14752663

[B26] Jacoby L. L. Lindsay D. S. Hessels S. (2003). Item-specific control of automatic processes: stroop process dissociations. *Psychon. Bull. Rev.* 10 638–644 10.3758/BF03196526 14620358

[B27] Jepma M. Nieuwenhuis S. (2011). Pupil diameter predicts changes in the exploration-exploitation trade-off: evidence for the adaptive gain theory. *J. Cogn. Neurosci.* 23 1587–1596 10.1162/jocn.2010.21548 20666595

[B28] Jepma M. Wagenmakers E.-J. Band G. P. H. Nieuwenhuis S. (2009). The effects of accessory stimuli on information processing: evidence from electrophysiology and a diffusion-model analysis. *J. Cogn. Neurosci.* 21 847–864 10.1162/jocn.2009.21063 18702584

[B29] Kahneman D. (1973). *Attention and Effort*. Englewood Cliffs, NJ: Prentice-Hall

[B30] Laeng B. Orbo M. Holmlund T. Miozzo M. (2011). Pupillary Stroop effects. *Cogn. Process.* 12 13–21 10.1007/s10339-010-0370-z 20865297 PMC3026931

[B31] Logan G. D. Zbrodoff N. J. (1979). When it helps to be misled: facilitative effects of increasing the frequency of conflicting stimuli in a Stroop-like task. *Mem. Cognit.* 7 166–174 10.3758/BF03197535

[B32] MacLeod C. M. (1992). The Stroop task: the “gold standard” of attentional measures. *J. Exp. Psychol. Gen.* 121 12–14 10.1037/0096-3445.121.1.12

[B33] Masson M. E. J. Loftus G. R. (2003). Using confidence intervals for graphically based data representation. *Can. J. Exp. Psychol.* 57 203–220 10.1037/h0087426 14596478

[B34] Mayr U. Awh E. Laurey P. (2003). Conflict adaptation effects in the absence of executive control. *Nat. Neurosci.* 6 450–452 10.1038/nn1051 12704394

[B35] Murphy P. R. O’Connell R. G. O’Sullivan M. Robertson I. H. Balsters J. H. (in press). Pupil diameter covaries with BOLD activity in human locus coeruleus. *Hum. Brain Mapp.* 10.1002/hbm.22466 PMC6869043 24510607

[B36] Murphy P. R. Robertson I. H. Balsters J. H O’Connell R. G. (2011). Pupillometry and P3 index the locus coeruleus–-noradrenergic arousal function in humans. *Psychophysiology* 48 1531–1542 10.1111/j.1469-8986.2011.01226.x 21762458

[B37] Nassar M. R. Rumsey K. M. Wilson R. C. Parikh K. Heasly B. Gold J. I. (2012). Rational regulation of learning dynamics by pupil-linked arousal systems. *Nat. Neurosci.* 15 1040–1046 10.1038/nn.3130 22660479 PMC3386464

[B38] Nieuwenhuis S. (2011). “Learning, the P3 and the locus coeruleus-norepinephrine system,” in *Neural Basis of Motivational and Cognitive Control* eds Mars R. Sallet J. Rushworth M. Yeung N. (London: Oxford University Press) 209–222

[B39] Nieuwenhuis S. de Geus E. J. Aston-Jones G. (2011). The anatomical and functional relationship between the P3 and autonomic components of the orienting response. *Psychophysiology* 48 162–175 10.1111/j.1469-8986.2010.01057.x PMC3797154 20557480

[B40] Nieuwenhuis S. Stins J. Posthuma D. Polderman T. Boomsma D de Geus E. (2006). Accounting for sequential trial effects in the flanker task: conflict adaptation or associative priming? *Mem. Cognit.* 34 1260–1272 10.3758/BF03193270 17225507

[B41] Notebaert W. Verguts T. (2007). Dissociating conflict adaptation from feature integration: a multiple regression approach. *J. Exp. Psychol. Hum. Percept. Perform.* 33 1256–1260 10.1037/0096-1523.33.5.1256 17924821

[B42] Phillips M. A. Szabadi E. Bradshaw C. M. (2000). Comparison of the effects of clonidine and yohimbine on spontaneous pupillary fluctuations in healthy human volunteers. *Psychopharmacology* 150 85–89 10.1007/s002130000398 10867980

[B43] Rouder J. N. Speckman P. L. Sun D. Morey R. D. Iverson G. (2009). Bayesian t tests for accepting and rejecting the null hypothesis. *Psychon. Bull. Rev.* 16 225–237 10.3758/PBR.16.2.225 19293088

[B44] Sanders A. F. (1983). Towards a model of stress and human performance. *Acta Psychol.* 53 61–97 10.1016/0001-6918(83)90016-1 6869047

[B45] Sara S. J. (2009). The locus coeruleus and noradrenergic modulation of cognition. *Nat. Rev. Neurosci.* 10 211–223 10.1038/nrn2573 19190638

[B46] Schmidt J. R. (2013). Questioning conflict adaptation: proportion congruent and Gratton effects reconsidered. *Psychon. Bull. Rev.* 20 615–630 10.3758/s13423-012-0373-0 23325703

[B47] Schmidt J. R. Besner D. (2008). The Stroop effect: why proportion congruent has nothing to do with congruency and everything with contingency. *J. Exp. Psychol. Learn. Mem. Cogn.* 34 514–523 10.1037/0278-7393.34.3.514 18444752

[B48] Schneider D. W. Logan G. D. (2005). Modeling task switching without switching tasks: a short-term priming account of explicitly cued performance. *J. Exp. Psychol. Gen.* 134 343–367 10.1037/0096-3445.134.3.343 16131268

[B49] Siegle G. J. Steinhauer S. R. Thase M. E. (2004). Pupillary assessment and computational modeling of the Stroop task in depression. *Int. J. Psychophysiol.* 52 63–76 10.1016/j.ijpsycho.2003.12.010 15003373

[B50] Silvetti M. Seurinck R. van Bochove M. E. Verguts T. (2013). The influence of the noradrenergic system on optimal control of neural plasticity. *Front. Behav. Neurosci.* 7:160 10.3389/fnbeh.2013.00160 PMC3826478 24312028

[B51] van Bochove M. Van der Haegen L. Notebaert W. Verguts T. (2013). Blinking predicts enhanced cognitive control. *Cogn. Affect. Behav. Neurosci.* 13 346–354 10.3758/s13415-012-0138-2 23242699

[B52] van Steenbergen H. Band G. (2013). Pupil dilation in the Simon task as a marker of conflict processing. *Front. Hum. Neurosci.* 7:215 10.3389/fnhum.2013.00215 PMC3665936 23754997

[B53] Vendrell P. Jungué C. Pujol J. Jurado M. A. Molet J. Grafman J. (1995). The role of prefrontal regions in the Stroop task. *Neuropsychologia* 33 341–352 10.1016/0028-3932(94)00116-7 7792000

[B54] Verguts T. Notebaert W. (2008). Hebbian learning of cognitive control: dealing with specific and nonspecific adaptation. *Psychol. Rev.* 115 518–525 10.1037/0033-295X.115.2.518 18426302

[B55] Verguts T. Notebaert W. (2009). Adaptation by binding: a learning account of cognitive control. *Trends Cogn. Sci.* 13 252–257 10.1016/j.tics.2009.02.007 19428288

[B56] Yu A. J. Dayan P. (2005). Uncertainty, neuromodulation, and attention. *Neuron* 46 681–692 10.1016/j.neuron.2005.04.026 15944135

